# Cu and Ni Co-Doped Porous Si Nanowire Networks as High-Performance Anode Materials for Lithium-Ion Batteries

**DOI:** 10.3390/ma16216980

**Published:** 2023-10-31

**Authors:** Can Mi, Chang Luo, Zigang Wang, Yongguang Zhang, Shenbo Yang, Zhifeng Wang

**Affiliations:** 1 School of Materials Science and Engineering, Hebei University of Technology, Tianjin 300401, China; 2 Key Laboratory for New Type of Functional Materials in Hebei Province, Hebei University of Technology, Tianjin 300401, China; 3 Collaborative Innovation Center for Vehicle Lightweighting, Hebei University of Technology, Tianjin 300401, China; 4 Hongzhiwei Technology (Shanghai) Co., Ltd., Shanghai 201206, China

**Keywords:** dealloying, doping, porous, lithium-ion battery, anode

## Abstract

Due to its extremely high theoretical mass specific capacity, silicon is considered to be the most promising anode material for lithium-ion batteries (LIBs). However, serious volume expansion and poor conductivity limit its commercial application. Herein, dealloying treatments of spray dryed Al-Si-Cu-Ni particles are performed to obtain a Cu/Ni co-doped Si-based anode material with a porous nanowire network structure. The porous structure enables the material to adapt to the volume changes in the cycle process. Moreover, the density functional theory (DFT) calculations show that the co-doping of Cu and Ni can improve the capture ability towards Li, which can accelerate the electron migration rate of the material. Based on the above advantages, the as-prepared material presents excellent electrochemical performance, delivering a reversible capacity of 1092.4 mAh g^−1^ after 100 cycles at 100 mA g^−1^. Even after 500 cycles, it still retains 818.7 mAh g^−1^ at 500 mA g^−1^. This study is expected to provide ideas for the preparation and optimization of Si-based anodes with good electrochemical performance.

## 1. Introduction

With the rapid development of the economy, portable electronic products and electric vehicles reveal increasingly high requirements for energy storage devices [1,2,3]. Lithium-ion batteries (LIBs) have received widespread attention as potential energy storage devices. However, due to the poor theoretical capacity of graphite electrodes (372 mA h g^−1^), the energy density of lithium-ion batteries is low, which cannot meet the market demand [4,5]. Therefore, it is an urgent task to develop high-capacity anode materials. Silicon (Si) has extremely high theoretical mass specific capacity (~4200 mA h g^−1^), low potential (<0.4 V vs. Li^+^/Li) and is abundant on Earth (the second most abundant element in the Earth’s crust) [6], making it the most promising anode material for the next generation of LIBs [7,8]. However, the two main drawbacks during lithiation/delithiation processes are significant volume changes (up to 300–400%) [9,10,11,12] and low intrinsic electrical conductivity (10^−5^ to 10^−3^ S cm^−1^) [13,14,15], which lead to dramatic structural damage and capacity loss that hinder the practical application of Si-based anodes.

To improve the structural stability and conductivity of Si, researchers from various countries have adopted various strategies, such as reducing the particle size of Si from the micrometer level to the nanometer level. In this situation, various forms of Si nanomaterials, including Si nanowires [16,17], Si nanotubes [18,19], and hollow Si nanospheres [20,21] with reasonable pore space, were synthesized to suppress cracking and fragmentation. Wang et al. prepared a coral-like network of Si branches with a one-dimensional nanowire structure that can accommodate significant volume changes during fast charging [22]. Due to the need for precision equipment and high synthesis costs in the synthesis process of existing Si nanomaterials, their production scale and practical application are limited [23]. Another way to improve the structural stability and conductivity of the Si anode is through carbon coating [24,25,26] or graphite mixing [27,28]. However, the addition of a large amount of carbon reduces the theoretical capacity and initial capacity of the Si-based anode [29]. In addition, another effective method to improve the conductivity and ion mobility of Si materials is to dope different elements [30], including boron (B) [31,32] and phosphorus (P) [33] into Si materials. B was doped by Chen et al. into a Si anode to obtain good initial Coulombic efficiency and long cycle stability [31]. In recent years, researchers have utilized different transition metals for doping/alloying/composite with Si anodes, which improve the structural stability and conductivity of materials and significantly improve their overall electrochemical performance [34].

Inspired by the above progress, Cu and Ni co-doped porous Si nanowire networks were obtained by dealloying spray dried Al-Si-Cu-Ni microspheres in this work. Dealloying refers to an etching process in which fewer valuable components are etched away from the precursor, and the remaining parts are self-assembled into various nano-/micro-porous structures. Some structural features of the precursor can be inherited and retained by the dealloying inheritance effect during the etching process [35,36]. At the same time, Al-Si-based powder synthesized by the spray drying method is a common spraying material used to repair aluminum, magnesium and their alloy parts. Both the spray drying method and the dealloying method involved in this paper are suitable for large-scale production [37,38]. Herein, it was found that the addition of Cu and Ni can improve the conductivity of Si-based anodes, enhance the electron transport rate in electrochemical reactions and the cycle stability of the battery. At 100 mAh g^−1^, a reversible capacity of 1092.4 mA g^−1^ can be maintained for 100 cycles. In addition, according to the density functional theory (DFT) calculations, it was revealed that the addition of copper and nickel promoted the adsorption of lithium atoms, and the adsorption energy was the highest when copper and nickel were co-doped at the same time, thus improved the electrochemical performance. This work has a positive impact on the development of a Si-based anode with low cost and high-performance.

## 2. Materials and Methods

### 2.1. Material Synthesis

**Figure 1:**
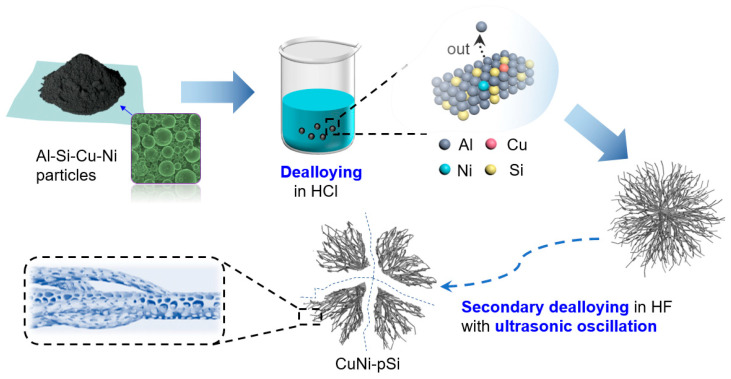
Schematic revealing the preparation process of CuNi-pSi.

The sample preparation process is shown in Figure 1. The initial precursor is Al-Si-Cu-Ni microsphere particles synthesized by spray drying method [39,40,41], which is obtained from China New Metal Materials Technology Co., Ltd., Beijing, China. The fabrication process of the final products mainly includes two chemical dealloying steps with/without ultrasonic oscillation. Firstly, the precursor particles were divided into five parts, and 1 g of Al-Si-Cu-Ni particles were dealloyed in 500 mL HF solution (0.02 M, 0.05 M, 0.1 M) for 4.5 h or in 300 mL HCl liquid (2.5% and 5%) for 5 days using five process parameters, respectively, to obtain the first step of dealloying products. The detailed conditions of the first step of dealloying are listed in Table 1. The related dealloying reaction formula for this step is shown as follows, revealing the etching of Al and the production of H_2_.

2Al + 6HCl = 2AlCl_3_ + 3H_2_ ↑ (1)

2Al + 6HF = 2AlF_3_ + 3H_2_ ↑ (2)

The second step of dealloying treatment was performed on 100 mg of the first step dealloying product in 50 mL of 5% HCl, 0.1 M HF, or 0.05 M HF, respectively, for 20 min, accompanied by ultrasonic oscillation. The detailed conditions of the second dealloying step are listed in Table 2.

All dealloyed samples (CuNi-pSi) were washed three times with anhydrous ethanol and then dried in a vacuum oven at 60 °C overnight for further characterization. The contrast material of porous Si (pSi) was also prepared by the same process as CuNi-pSi through dealloying of Al_90_Si_10_ microsphere particles.

### 2.2. Material Characterization

The Cu Kα X-ray diffractometer (XRD, D8 Discover, Bruker, Karlsruhe, Germany) was used to study the phase structure of the samples at 2θ, in the range of 10–90°. The morphology of the samples was observed by scanning electron microscopy (SEM, JSM-6700F, JEOL, Tokyo, Japan) and transmission electron microscopy (TEM, JEM 2100F, JEOL, Tokyo, Japan). The Brunauer Emmett Teller (BET) method and the Barrett Joyner hallenda (BJH) method were used to analyze the specific surface area and pore size distribution of the materials, respectively. To further detect the elemental composition and valence states of the sample, X-ray photoelectron spectroscopy (XPS, V-Sorb 2800P, Beijing, China) was used. Raman spectra were measured in the wavenumber range of 100~2000 cm^−1^.

### 2.3. Electrochemical Measurements

The active materials (CuNi-pSi), conductive agent and binder were mixed at a weight ratio of 7:2:1, and then deionized water was added for grinding to make a uniform slurry and coated on the copper foil. After drying at 60 °C for 12 h, the original tablets were cut into 10 mm, and the active substance loaded on each electrode was about 1.1 mg. The electrolyte is 1 M LiPF_6_ in the mixture of ethylene carbonate (EC) and dimethyl carbonate (DMC) (volume ratio 1:1), and lithium foil is used as the counter electrode. The charge/discharge measurements of the assembled battery were carried out by the NEWARE CT-4000 battery testing system at room temperature. The cyclic voltammetry (CV) curves and EIS spectra were measured on the electrochemical workstation (Princeton: Versa STAT 4).

### 2.4. DFT Calculations

Based on DFT, all our calculations are performed using DS-PAW software (HZWTECH, version 2022A, Shanghai, China) integrated into the Device Studio program [42,43]. The pure Si surface was constructed by the 2 × 2 supercell of Si (111) of three layers of atoms. The surface of CuNi-pSi is obtained by replacing Si atoms with Cu and Ni atoms on the surface of Si. In order to avoid the interaction between adjacent configurations, it is necessary to apply a vacuum region of 15 Å. In addition, the energy cutoff was chosen to be 500 eV, and the Monkhorst-Pack of k-points with a 3 × 3 × 1 grid was used to sample the Brillouin zone. The convergence criteria for energy and force calculations are set at 10^−6^ EV and 0.05 EV Å.

## 3. Results and Discussion

**Figure 2:**
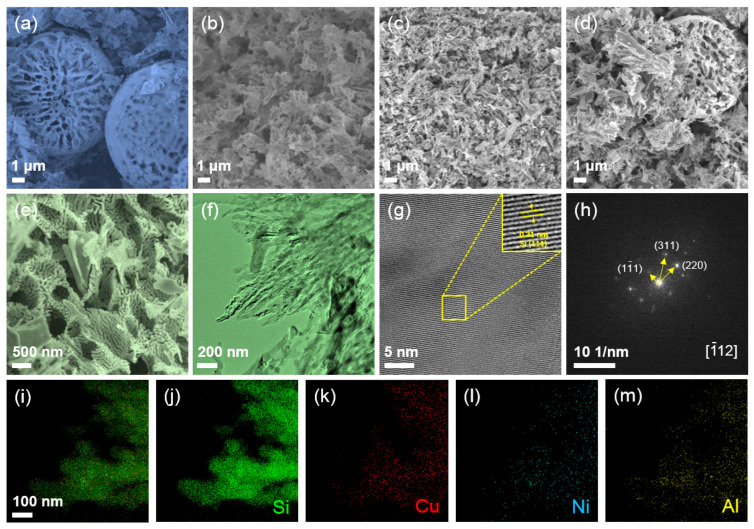
SEM images of the Al-Si-Cu-Ni precursor after the first step of dealloying (Sample-5) (**a**) and the second step of dealloying: (**b**) Sample-6, (**c**) Sample-7, and (**d**,**e**) CuNi-pSi; (**f**) TEM image, (**g**) HRTEM image, and (**h**) SAED patterns of the CuNi-pSi material; (**i**–**m**) elemental mapping of the CuNi-pSi product: (**i**) Mix, (**j**) Si, (**k**) Cu, (**l**) Ni, and (**m**) Al.

Figure S1a shows the morphology of the Al-Si-Cu-Ni particles with a symmetrical and smooth spherical structure (average diameter of about 10 μm). Figure S1b–f reveals a backscatter electron image (BSEM) and corresponding EDS mapping of the precursor particles, indicating a uniform distribution of Al, Si, Cu, and Ni elements. The atomic ratio of Al:Si:Cu:Ni is about 89.53:10.26:0.08:0.13 based on EDS results (Figure S1g). The SEM images of the samples after the first dealloying step are shown in Figure S2. It can be seen from Figure S2a–d that the corrosion toward sample-1 to sample-4 is insufficient, and rare pores can be formed in these processes. In stark contrast to these samples, Figure S2e and Figure 2a present an obviously porous morphology, implying that the current etching conditions (sample-5) are effective for the formation of porous networks. Because the precursor belongs to a typical hypoeutectic Al–Si based alloy, it mainly contains network eutectic and a large amount of Al. When it is etched under suitable conditions, the aluminum phase in the precursor will be removed, leaving only the eutectic network skeleton. In order to obtain a higher specific surface area, the second step of the dealloying treatment was performed (Figure 2b–e), accompanied by ultrasonic treatment. In this way, most of the Al elements in eutectic nanowires are further etched to create porous nanowires (Figure 2f). At the same time, through ultrasonic vibration, the microspheres collapse into porous network fragments, thus eliminating the internal uncorroded zone. It can be found that among the three different second-step etching processes (Figure S3 and Figure 2e), the experimental parameter of 0.05 M HF and the resulting porous morphology is the optimum. In this situation, narrow ligaments and uniform network structures can be formed. The large number of pores during the cycling process can effectively adapt to the volume changes in the electrode [44,45], which is conducive to electrolyte permeation and rapid ion transport, while the nanowire network is conducive to rapid electron transfer [46]. The SEM images of pSi are shown in Figure S4, showing a similar morphological characteristic as CuNi-pSi.

Figure 2g uncovers the HR–TEM image of the sample after two dealloying processes, showing the crystal plane spacing of 0.31 nm, which is consistent with the Si (1–11) plane [47]. The selected regional electron diffraction (SAED) pattern (Figure 2h) [48] further reveals the Si [−112] zone axis [31]. The EDS mapping shown in Figure 2i–m presents the uniform distribution of Si, Cu, Ni, and Al elements. The atomic ratio of Al:Si:Cu:Ni of the local nanowire based on TEM mapping is about 3.38:95.69:0.46:0.47 (Figure S5) and the residual Al content is similar to that of pSi (Figure S6). Trace doping of Cu and Ni elements may improve electronic conductivity and reaction kinetics during the charge–discharge cycle [49,50], while a small amount of Al can increase conductivity, thereby increasing the lithiation kinetics [51].

**Figure 3:**
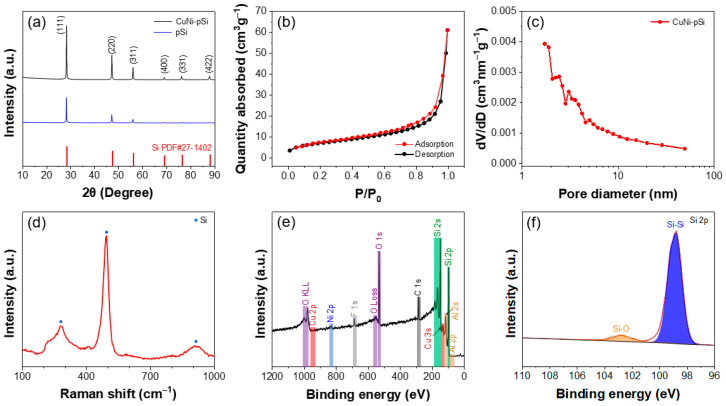
(**a**) XRD patterns of CuNi-pSi and pSi. N_2_ adsorption-desorption isotherms of CuNi-pSi (**b**) and corresponding pore-size distribution (**c**). (**d**) Raman spectra of CuNi-pSi, red line: the measured curve. XPS spectra of CuNi-pSi: (**e**) XPS survey spectra, black line: the measured curve, color areas: the peaks of different elements; (**f**) high-resolution core-level spectra of Si 2p, black line: base line, red line: the fitting curve, color areas: different peaks of silicon.

The X-ray diffraction (XRD) patterns of the dealloyed CuNi-pSi are displayed in Figure 3a. All the peaks can be indexed to cubic Si (JPCDS 27-1402) [33]. Compared with peak intensities of pSi, that of CuNi-pSi is higher, indicating that the crystallinity of CuNi-pSi is higher. The porous structure of the CuNi-pSi sample was further disclosed by the N_2_ adsorption–desorption isotherm curves (Figure 3b). The type III curve with an H3-type hysteresis loop is revealed, indicating the presence of a certain content of mesopores in the product [52]. The specific surface area of the material is about 25.08 m^2^ g^−1^, and the pore size is mainly between 1.7 and 10 nm (Figure 3c). These pores are able to facilitate the permeation of electrolytes and allow for more complete lithiation reactions. Raman spectra of the CuNi-pSi sample are shown in Figure 3d. The dominating peaks at 495 cm^−1^ and the two weak peaks at 280 cm^−1^ and 925 cm^−1^ are typical signals of Si. Compared to the standard silicon peak of 510 cm^−1^, the peak of CuNi-pSi shifts to 495 cm^−1^. This shift is due to the stress generation surrounding the Si network after the co-doping of Cu and Ni, resulting in a structural disorder in Si [31]. The elemental valence state of the CuNi-pSi is further investigated by XPS. A typical XPS survey spectrum confirms the presence of Si, Cu, Ni and Al (Figure 3e). Si 2p XPS spectra are displayed in Figure 3f. The peak at 98.74 eV belongs to the Si−Si bond, while weak peaks with the binding energy of 102.84 eV can be ascribed to the Si−O bond [53], indicating the presence of trace Si oxides on the sample surface. However, there is no obvious XRD peak of silicon oxide in Figure 3a, meaning that the content of silicon oxide is very small [54]. The XPS spectra of Ni 2p, Cu 2p, O 1s and Al 2p are shown in Figure S7a–d, respectively. The minimum element proportion threshold at which the XPS device can detect an element signal is about 5%. Because the ratio of Cu and Ni elements in CuNi-pSi is less than 5%, there are weak characteristic peaks of Cu 2p and Ni 2p in the XPS fine spectrum. Moreover, the residual Al exists in the form of Al^3+^ due to unavoidable surface oxidation, while O presents an obvious M–O bond (M refers to Si, Al, Ni and Cu in this work).

**Figure 4:**
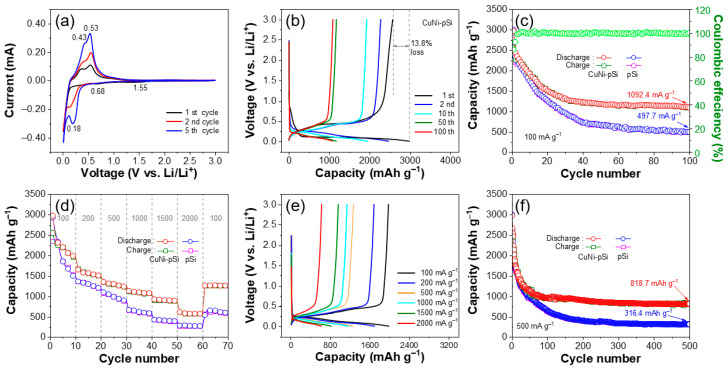
(**a**) CV curves of the CuNi-pSi at 0.1 mV s^−1^. (**b**) Charge–discharge curves of the CuNi-pSi anode at the current density of 100 mA g^−1^. (**c**) Cycling performance of the two anodes at the current density of 100 mA g^−1^. (**d**) Rate performance of the two anodes at various current densities. (**e**) Charge–discharge profiles of the CuNi-pSi anode at various current densities. (**f**) Cycling performance of the two anodes at the current density of 500 mA g^−1^ (the batteries are firstly activated at 100 mA g^−1^ for three cycles).

To better elucidate the effect of trace doping of Cu and Ni on electrochemical performance, pSi (Figure S4a,b) was also obtained by the same preparation process and tested as a control sample of CuNi-pSi. Thus, a series of electrochemical tests were conducted. Figure 4a shows the CV curves of the CuNi-pSi electrode in the first five cycles. In the initial discharge process, two broad cathode peaks appeared at 1.55 V and 0.68 V due to the formation of the solid electrolyte interface (SEI). In the charging process, the anode peaks located at around 0.43 V and 0.53 V correspond to the Li separation process from lithium silicide. In the subsequent cycle, the two broad cathode peaks disappeared, indicating that the SEI layer generated on the electrode surface quickly reached stability [55,56]. The cathode peak at around 0.18 V can be attributed to the lithiation of Si. The reversible discharging–charging process of Si can be expressed as the following reaction [2]:

Li_x_Si ⇆ Si + xLi^+^ + xe^−^ (3)

Meanwhile, due to the activation process of Si, an increase in CV peak intensity was observed in the initial few cycles, which can also be observed in previously reported Si-based anode materials [29]. In addition, the curve shows that when the scanning voltage (vs. Li^+^/Li) exceeds 2 V, the capacity provided by the battery accounts for a very small proportion of the total capacity. Comparing the CV curves of CuNi-pSi and pSi (Figure S8), the peak positions are basically consistent, and the CuNi-pSi displays a larger integrated area than the pSi, indicating that CuNi-pSi has better electrochemical activity and excellent reversible lithium storage capacity.

Figure 4b shows the constant current charge–discharge curves of CuNi-pSi at 100 mA g^−1^. All voltage platforms in the discharge and charging curves correspond well to the cathode and anode peaks in the CV curve. The discharge plateau in the first cycle is relatively low, which is caused by the first lithiation of Si, representing the stable formation of the SEI layer. The initial discharge capacity of the CuNi-pSi electrode is 2972 mAh g^−1^, while the charging capacity is 2560 mAh g^−1^, which is equivalent to a capacity loss of 13.8%. The main reason for the capacity loss in the first cycle is the formation of the SEI layer. As the number of cycles increases, the curve gradually moves to the left, indicating a gradual decrease in capacity. The discharge–charge platform is well maintained after 100 cycles, indicating that the electrochemical process of CuNi-pSi is relatively stable.

The cyclic performance of sample-1 to sample-7 at a current density of 100 mA g^−1^ is shown in Figure S9. It can be clearly seen that after the first dealloying step, sample-5 exhibits the best electrochemical properties among the first five samples, which is related to its good porous structure. In view of the advantages of the three-dimensional structure and electrochemical properties, it is reasonable to use sample-5 as a precursor in the second step of corrosion. The cyclic performance of CuNi-pSi and pSi at 100 mA g^−1^ is shown in Figure 4c. It can be seen that the capacity of CuNi-pSi rapidly decays during the first 30 cycles and gradually stabilizes thereafter. During the initial cycles, the formation of SEI membranes leads to the consumption and loss of some active lithium, resulting in a decrease in capacity. The capacity fade may be recovered through a newly reported reserve lithium-ion battery strategy [57]. Moreover, the porous nanowire network structure provides room to adapt to the volume expansion of Si and eventually reach a stable state. At 100 mA g^−1^, CuNi-pSi provides a reversible capacity of 1092.4 mA g^−1^ after 100 cycles, while pSi rapidly decays to below 1000 mA g^−1^ after only 30 cycles. It is evident that the CuNi-pSi porous nanowire networks doped with Cu and Ni exhibit more stable cycling performance compared with sample-1 to sample-7 and pSi. One possible reason is that the slow electrochemical kinetics of Si leads to a large amount of lithium detained in the Si anode [58], while the addition of Cu and Ni accelerates the electrochemical kinetics of the Si-based anode.

Figure 4d shows the rate performance of the two anodes at current densities from 100 to 2000 mA g^−1^. The reversible capacities of the CuNi-pSi anode at 100, 200, 500, 1000, 1500 and 2000 mA g^−1^ are 2203.54, 1569.75, 1301.36, 1098.33, 906.19 and 575.05 mAh g^−1^, respectively. As the current density restores to 100 mA g^−1^, the discharge capacity returns to 1264 mAh g^−1^, indicating that CuNi-pSi has good reversibility. Accordingly, the pSi anode maintains a capacity of 274 mAh g^−1^ at 2000 mA g^−1^. When the current density is restored to 100 mA g^−1^, only 602.15 mAh g^−1^ reversible capacity can be maintained. Moreover, CuNi-pSi has a higher capacity compared to pSi at different current densities, uncovering that CuNi-pSi has a better rate performance and reversibility. The comparison of the rate capability of the CuNi-pSi and pSi anode can be more clearly observed in the charge–discharge curve at different current densities shown in Figure 4e and Figure S8b. It is obvious that as the current density gradually increases from 100 mA g^−1^ to 2000 mA g^−1^, the curves gradually shift to the left, and the reversible capacity decreases with the increase in current density. The distance between the charge and discharge curves of CuNi-pSi at different current densities is smaller than that of pSi, indicating that CuNi-pSi has better rate performance.

Figure 4f shows the cycling performance of CuNi-pSi and pSi anodes. The batteries are firstly activated at 100 mA g^−1^ for three cycles and then cycled at 500 mA g^−1^ for four hundred and ninety-seven cycles. CuNi-pSi maintains a reversible capacity of 818 mAh g^−1^ after 500 cycles, while pSi experiences severe capacity decay, maintaining only 316.4 mAh g^−1^ after 500 cycles. The excellent cycling performance of CuNi-pSi can be attributed to the better conductivity of the Si-based anode due to the addition of Cu and Ni. A small amount of Cu and Ni atoms are embedded in Si, suppressing the volume and morphology changes in the silicon anode, reducing the drastic volume changes in silicon, preventing the pulverization of silicon anodes, and maintaining the nanowire network structure [59].

**Figure 5:**
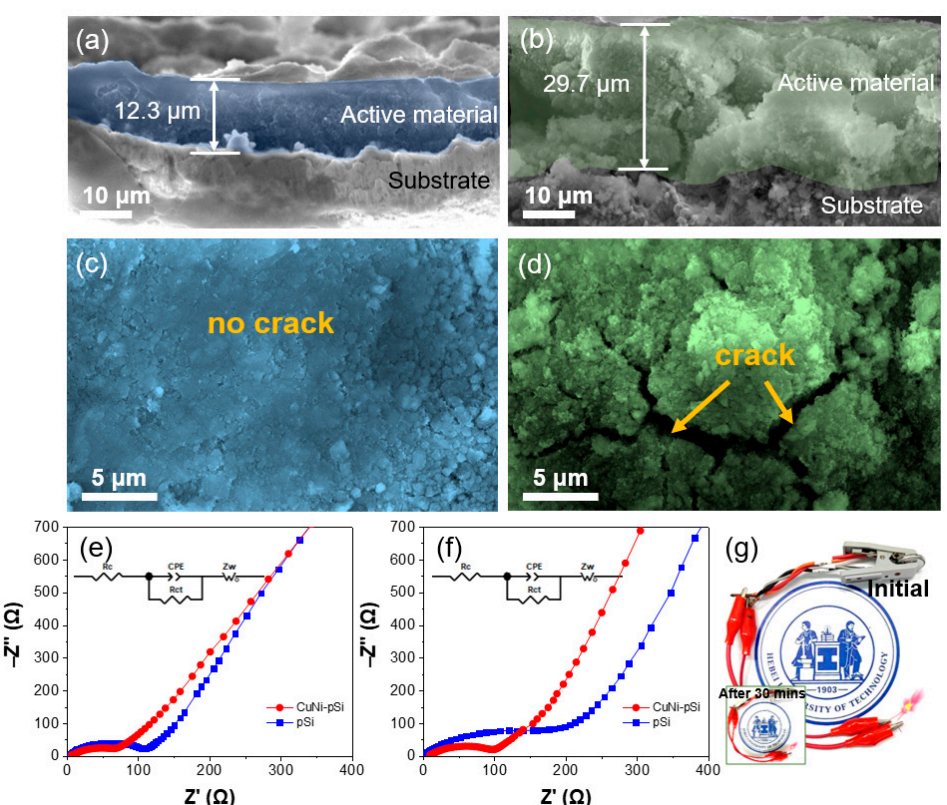
Cross-sectional and typical top-view SEM images of (**a**,**c**) CuNi-pSi and (**b**,**d**) pSi after cycling at the current density of 100 mA g^−1^ for 100 cycles. EIS spectra equivalent circuit (inset) of the CuNi-pSi and pSi anodes: (**e**) Fresh, (**f**) After cycling at the current density of 100 mA g^−1^ for 100 cycles. (**g**) LED bulb powered by the battery with CuNi-pSi anodes.

To further understand the electrochemical behavior of the two anodes, the electrode morphology after cycling for 100 cycles at 100 mA g^−1^ was characterized. Cross-sectional and typical top-view SEM images of the CuNi-pSi electrode after cycling are shown in Figure 5a,c. During cycling, due to changes in electrode volume, some porous Si nanowires gradually merged together, but the overall structural integrity of the electrode remained good without fracture. The thickness of the active materials is about 12.3 nm. Figure 5b,d presents the cross-sectional and top-view SEM images of the pSi electrode after cycling. Obvious cracks can be observed, and the thickness of the active materials is about 29.7 μm, which is more than twice as thick as that of CuNi-pSi. This proves that the CuNi-pSi electrode can adapt to the volume change in the process of cycling and can keep a relatively low expansion rate. Figure 5e shows the EIS diagram and equivalent circuit (inset) of two fresh samples. The EIS curve shows a semicircle in the high-frequency region and a slope line in the low-frequency region, corresponding to the charge transfer resistance and ion diffusion resistance, respectively [60]. The resistance of CuNi-pSi is smaller than that of pSi, which means that the addition of copper and nickel increases the conductivity of the anode. The EIS diagram and equivalent circuit of the sample after cycling for 100 cycles are shown in Figure 5f. Compared with the pSi anode, the resistance of the CuNi-pSi anode increases less after cycling. This is because the co-doping of Cu and Ni can reduce the huge stress caused by the volume change during the cycling process to ensure the integrity of the electrode so that the anode presents better cycle stability. Furthermore, during the cycling process, the expansion rate of Al after lithium absorption (94%) is lower than that of Si (280%), which can effectively prevent the lattice expansion of Si and improve its performance as an anode material for lithium-ion batteries [51]. As shown in Figure 5g, in order to investigate the usability of the Si anode, the battery was connected to a light bulb, which emitted bright light and did not significantly darken after 30 min, representing the enormous application prospects of the CuNi-pSi anode.

**Figure 6:**
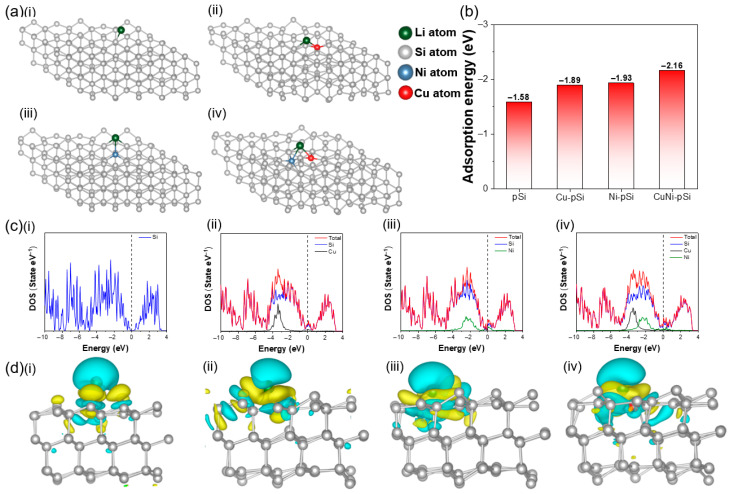
(**a**) optimized the adsorption of Li, (**b**)calculated the adsorption energy of Li on the surface, (**c**) projected DOS (PDOS) and (**d**) differential charge density of (**i**) pSi, (**ii**) Cu-pSi, (**iii**) Ni-pSi and (**iv**) CuNi-pSi.

To further analyze the effects of Cu and Ni doping on lithium-ion reaction kinetics and material conductivity, DFT calculations were performed. Four different models were constructed, including pure Si, single Cu-doped Si (Cu-pSi), single Ni-doped Si (Ni-pSi), and Cu/Ni co-doped Si (CuNi-pSi). The models of four kinds of materials after adsorbing lithium-ions are shown in Figure 6a. The calculation formula of adsorption energy is [61]:

E_ad_ = E_SiLi_ − E_Si_ − E_Li_ (4)

The adsorption energies of pSi, Cu-pSi, Ni-pSi, and CuNi-pSi are −1.58, −1.89, −1.93, and −2.16 eV, respectively (Figure 6b). The lower adsorption energy after doping indicates that the addition of Cu and/or Ni can promote the adsorption of lithium atoms. The doping effect of Ni toward Si is slightly higher than that of Cu. Moreover, when Cu and Ni are co-doped into Si, the lowest adsorption energy and best absorption efficiency can be obtained. Figure 6c shows the projected density of state (PDOS) of pSi, Cu-pSi, Ni-pSi, and CuNi-pSi; the PDOS diagram of pure Si shows a clear bandgap (0.3 eV), which is a classic semiconductor characteristic with low conductivity. In contrast, the bandgap of the doped PDOS pattern disappears, indicating an improvement in material conductivity. Near the Fermi levels, the contribution of Ni is higher than that of Cu, which is consistent with the adsorption energy, confirming that the doping of copper and nickel can regulate the electronic structure and further promote the storage of Li^+^. To better understand the influence of Cu and Ni doping on the electrochemical performance, the differential charge densities of pSi, Cu-pSi, Ni-pSi, and CuNi-pSi are calculated, as shown in Figure 6d. The figure clearly shows the charge transfer of Si materials after the adsorption of lithium atoms. The yellow and cyan areas represent the increase and decrease in charge density, respectively. It can be seen from the figure that the addition of Cu and Ni makes a stronger charge transfer of lithium. In order to quantify the amount of electron transfer, Bader charge analysis (Table S1) was conducted. The results showed that the electron numbers transferred by lithium atoms in Si, Cu-pSi, Ni-pSi, and CuNi-pSi were 0.8653, 0.8696, 0.8748, and 0.8821 e, respectively. CuNi-pSi presents the highest charge transfer amount, and the charge transfer amount of Ni-pSi is higher than that of Cu-pSi, which corresponds to the adsorption energy, indicating that Ni doping plays a slightly greater contribution. From the above data, it can be reasonably inferred that the doping of copper and nickel enables Si-based anodes to achieve superior Li capture ability, enhance the surface reactivity and conductivity of the anode, and thus achieve excellent cycle life and rate performance. Moreover, CuNi-pSi contains a small amount of aluminum, which can shift the Fermi level guide band and valence band, increasing the conductivity of Si.

In many dealloying reactions, the aluminum element is not completely removed. A small amount of its residue is generally considered not to have much effect on the electrochemical properties of LIBs. On the one hand, residual aluminum may cause capacity reduction, but the impact is limited. The distribution of these residual aluminum elements on the silicon ligament is relatively uniform, while not all aluminum is enriched on the surface of silicon. Only a small amount of aluminum is located on the surface of the ligament, causing limited capacity reduction by irreversible Al–Li alloys, while most aluminum is encased in silicon ligaments and does not react with lithium directly. On the other hand, trace amounts of aluminum may increase the electrical conductivity of silicon, thereby improving electrode performance. According to the above discussion, the positive and negative effects of aluminum on the material cancel each other out and ultimately do not have much impact on the electrochemical properties of the material.

Table 3 [48,56,59,62,63,64,65,66,67,68] shows the comparison of the electrochemical performance of CuNi-pSi and the previously reported Si-based anode. It can be found that CuNi-pSi displays excellent lithium storage performance in different studies. The main problem of the Si-based anode is that the continuous insertion and extraction of Li ions in the cycle process produce large mechanical stress in the Si material, resulting in material pulverization and electrode cracking. Moreover, a large number of volume fluctuations also make the fragile SEI unbearable, resulting in the unstable and continuous growth of the SEI layer. The CuNi-pSi presents good performance, which can be attributed to the following reasons. Firstly, CuNi-pSi has a porous nanowire network structure with rich porosity, which provides sufficient space for the expansion of the Si anode. Secondly, the addition of Cu and Ni can provide support to deal with the mechanical stress in the cycle process and maintain the integrity of the electrode structure. Thirdly, Cu and Ni are conductors, which can improve the conductivity of the Si anode. They not only improve the cycle stability of the battery but also improve the rate performance. In this paper, based on the structure adjustment strategy of a two-step dealloying process, Cu and Ni co-doping have been successfully implemented, which has a positive impact on the development of low-cost Si-based anodes.

Although the current preparation method uses acid and the processing time is long, which makes the process seem less green and time-consuming, there are many opportunities to optimize the existing process and reduce the use of acid and processing time. In addition, the technological route in this paper still has some advantages, as follows. Firstly, the samples were prepared by spray drying and dealloying methods, both of which are suitable for large-scale production. Secondly, the products fabricated by the related process present a good three-dimensional skeleton structure and rich hierarchical porous structure. The pores in the nanowire network structure and the pores on the nanowire are conducive to electrolyte permeation, allowing for more complete lithiation reactions and adapting to the significant volume changes in Si during cycling. Nanowire networks can establish rich three-dimensional channels, reduce ion transport distance, enhance reaction kinetics, and improve the electrochemical performance of Si anodes. As a result, the material shows promising electrochemical performance.

## 4. Conclusions

In summary, Cu and Ni co-doped porous Si nanowire networks were prepared by a two-step dealloying method and were used as Li-ion battery anodes, revealing an excellent electrochemical performance of 1092.4 mAh g^−1^ after 100 cycles at 100 mA g^−1^. The reversible capacity of 818.7 mAh g^−1^ can be retained even after 500 cycles at 500 mA g^−1^. DFT calculations show that the addition of copper and nickel can improve the adsorption capacity of the anode toward Li and can improve the conductivity and electrochemical reactivity of the Si material. The combination of a porous nanowire network structure and Cu/Ni co-doping contributes to good lithium storage performance, which is expected to promote the exploration of Si-based anode with low cost and high-performance.

## Figures and Tables

**Table 1:** Treatment conditions of the first step of dealloying process.

Precursor	Corrosive Solutions	Corrosion Time	Sample Name
Al-Si-Cu-Ni particles 1.0 g	0.02 M HF, 500 mL	4.5 h	Sample-1
	0.05 M HF, 500 mL		Sample-2
	0.1 M HF, 500 mL		Sample-3
	2.5% HCl, 300 mL	5 days	Sample-4
	5% HCl, 300 mL		Sample-5

**Table 2:** Treatment conditions of the second step of dealloying process.

Precursor	Corrosive Solutions	Corrosion Time	Sample Name
Sample-5 100 mg	5% HCl, 50 mL	20 min	Sample-6
	0.1 M HF, 50 mL		Sample-7
	0.05 M HF, 50 mL		CuNi-pSi

**Table 3:** The comparison of electrochemical performance of Si-based anodes.

Anode Material	Current Density (mA g^−1^)	Cycle Number	Reversible Capacity (mAh g^−1^)	Reference
Si	100	100	1015	[62]
Si	100	100	980	[61]
Si	500	200	993	[56]
Si/C-P	200	200	883.4	[59]
Si/SiO_x_	200	53	492	[64]
Si/N	200	150	690	[65]
Si/C	210	200	1110	[48]
Si/C	100	100	934	[66]
SiOC	500	300	501	[67]
Mg-Si/SiO_x_	150	100	490	[68]
CuNi-pSi	100	100	1092.4	This work
	500	500	818.7	

## Data Availability

The data presented in this study are available upon request from the corresponding author.

## Supplementary Materials

The following supporting information can be downloaded at: https://www.mdpi.com/article/10.3390/ma16216980/s1; Figure S1: Morphology and EDS of Al-Si-Cu-Ni particles; Figure S2: SEM images after the first step of dealloying; Figure S3: SEM images after the second step of dealloying; Figure S4: SEM images of pSi; Figure S5: EDS analysis of CuNi-pSi; Figure S6: EDS analysis of pSi; Figure S7: XPS spectra of CuNi-pSi; Figure S8: CV curves and Charge-discharge profiles at various current densities of pSi; Figure S9: Cycling performance of contrast materials. Table S1: Bader charge for pSi, Cu-pSi, Ni-pSi and CuNi-pSi.
