# Supplementary material for: Cu and Ni Co-Doped Porous Si Nanowire Networks as High-Performance Anode Materials for Lithium-Ion Batteries
Source: Materials (Basel). 2023 Oct 31;16(21):6980. doi: 10.3390/ma16216980 (PMC10650621; doi:10.3390/ma16216980)
Supplement: Supplementary file 1 [file materials-16-06980-s001.zip › materials-2621780-supplementary.pdf]

Supporting information

Cu and Ni co-doped porous Si nanowire networks as high-performance anode materials for lithium-ion batteries

Can Mi [1,2,3,†], Chang Luo [1,†], Zigang Wang [1], Yongguang Zhang [1,*], Shenbo Yang [4] and Zhifeng Wang [1,2,3,*]

[1] School of Materials Science and Engineering, Hebei University of Technology, Tianjin 300401, China
[2] Key Laboratory for New Type of Functional Materials in Hebei Province, Hebei University of Technology, Tianjin 300401, China
[3] Collaborative Innovation Center for Vehicle Lightweighting, Hebei University of Technology, Tianjin 300401, China
[4] Hongzhiwei Technology (Shanghai) Co. Ltd., Shanghai 201206, Shanghai, China
* Correspondence: yongguangzhang@hebut.edu.cn (Y.Z.); wangzf@hebut.edu.cn (Z.W.); Tel.: +86-22-60202006 (Z.W.)
† These authors contributed equally to this work.

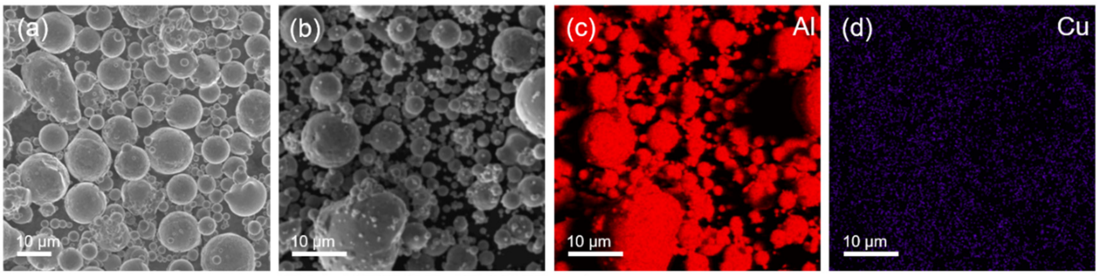

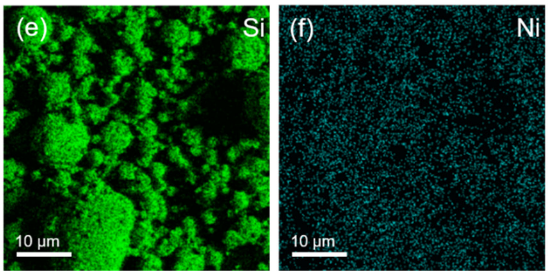

(g)

Element	Weight%	Atomic%
Al K	88.92	89.53
Si K	10.61	10.26
Ni K	0.28	0.13
Cu K	0.19	0.08

Figure S1. SEM (a) and BSEM (b) of Al-Si-Cu-Ni particles. EDS mapping (c-f) and elemental ratio (g) of Al-Si-Cu-Ni particles.

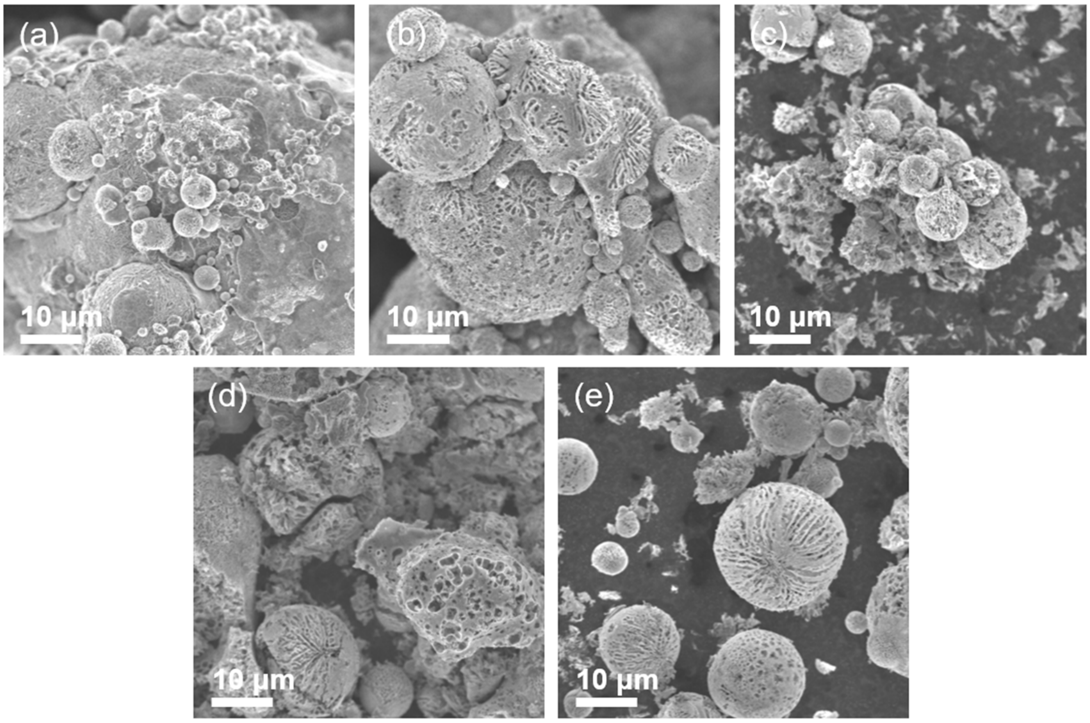

Figure S2. SEM images after the first step of dealloying. (a) Sample-1, (b) Sample-2, (c) Sample-3, (d) Sample-4 and (e) Sample-5.

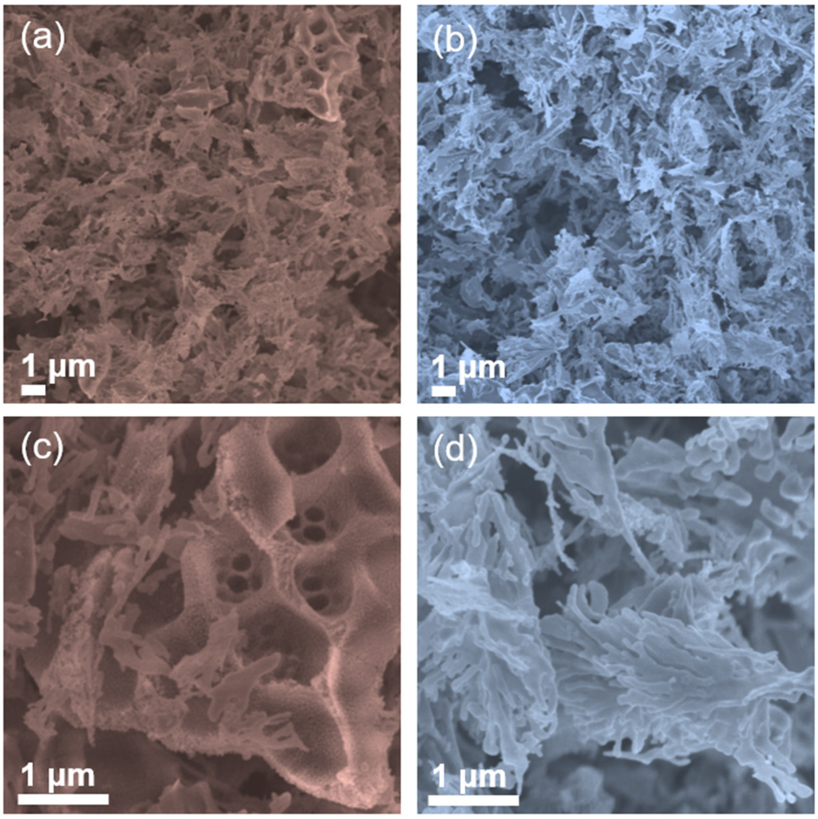

Figure S3. SEM images after the second step of dealloying. (a,c) Sample-6 and (b,d) Sample-7.

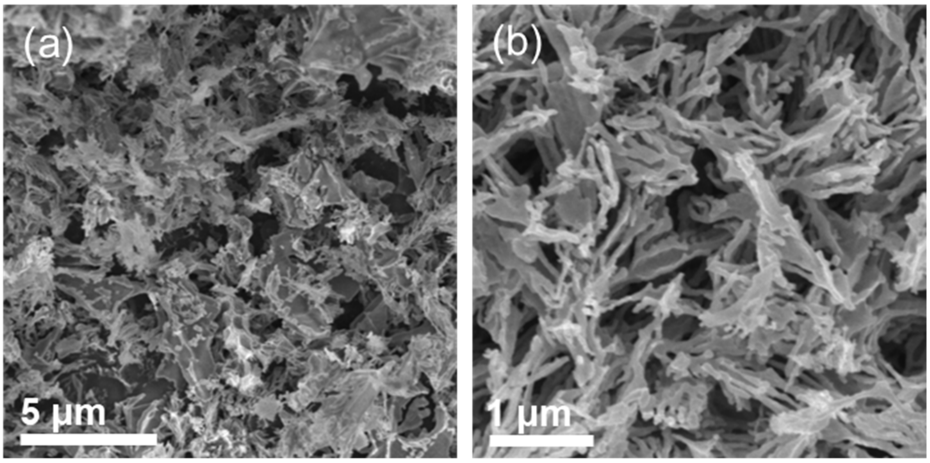

Figure S4. SEM images of (a,b) pSi.

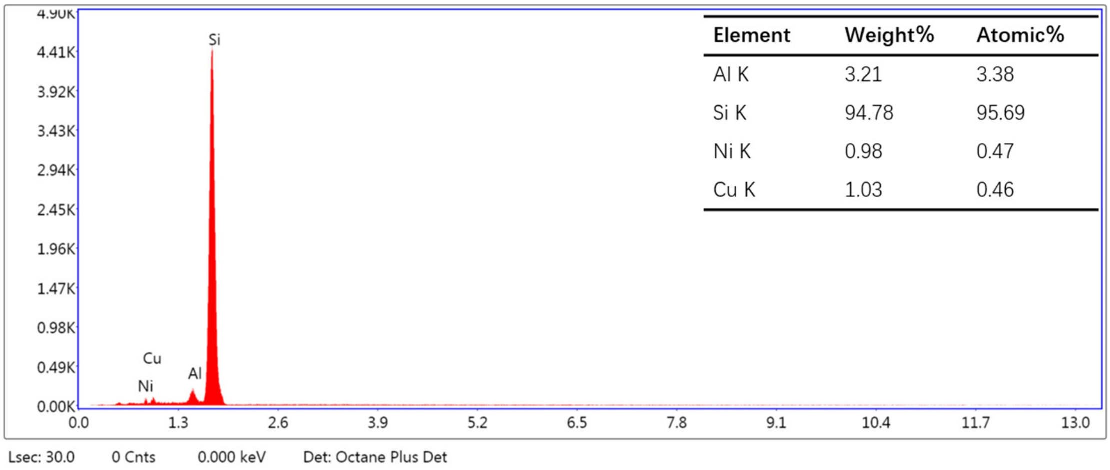

Element	Weight%	Atomic%
Al K	3.21	3.38
Si K	94.78	95.69
Ni K	0.98	0.47
Cu K	1.03	0.46

Figure S5. EDS analysis of CuNi-pSi.

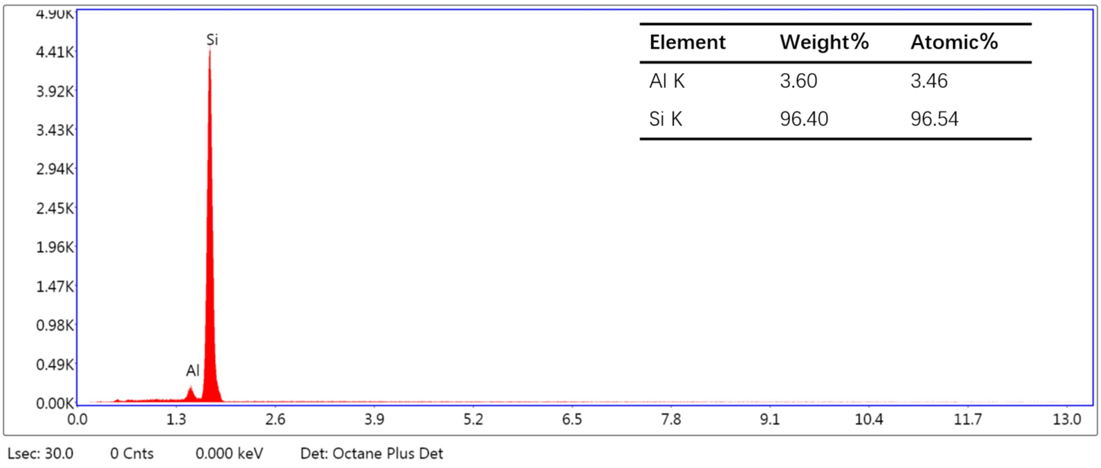

Figure S6. EDS analysis of pSi.

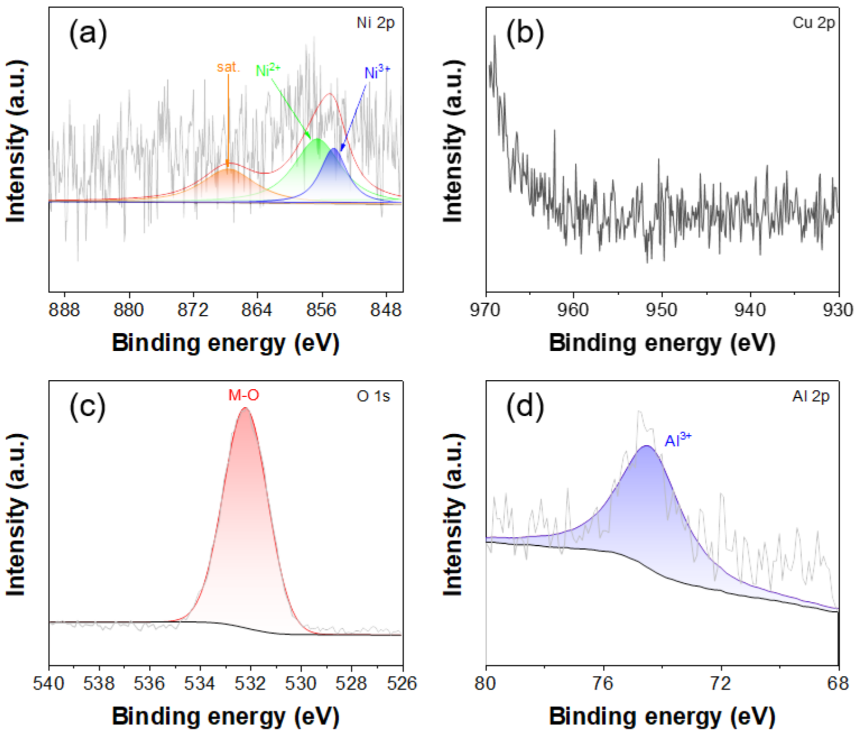

Figure S7. XPS spectra of CuNi-pSi. High-resolution core-level spectra of (a) Ni 2p , (b) Cu 2p, (c) O 1s and (d) Al 2p.

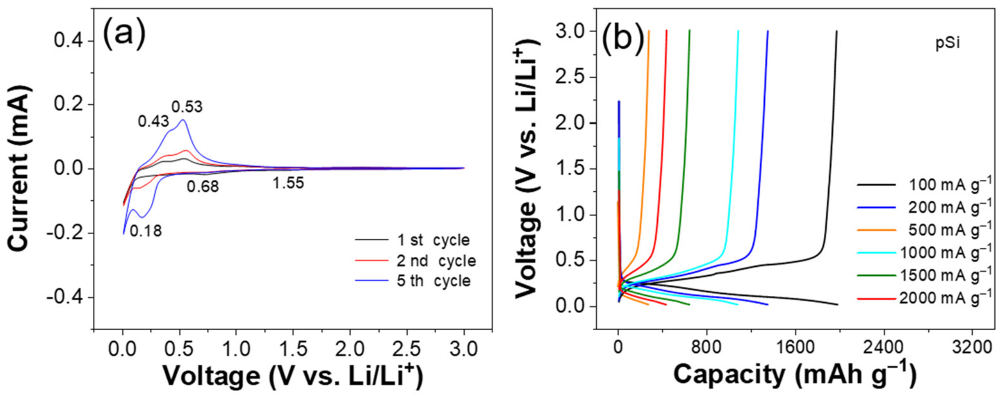

Figure S8. (a) CV curves of the pSi at 0.1 mV s^{-1}. (b) Charge-discharge profiles of the pSi anode at various current densities.

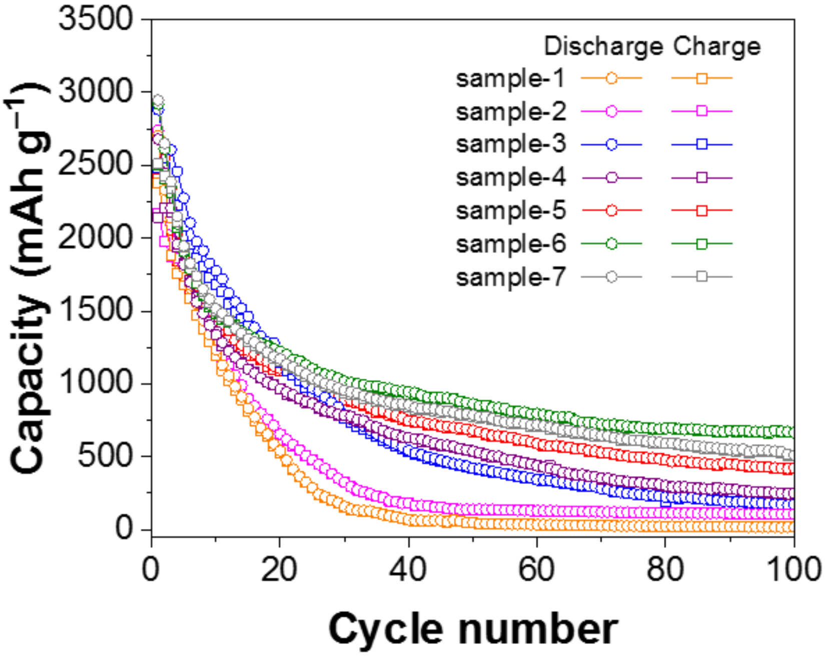

Figure S9. Cycling performance of contrast materials at a current density of 100 mA g^{-1}.

Table S1. Bader charge for pSi, Cu-pSi, Ni-pSi and CuNi-pSi.

Element \ Material / Charge	pSi	Cu-pSi	Ni-pSi	CuNi-pSi
**Li**	0.8653	0.8696	0.8748	0.8821
**Cu**		0.0746		0.1606
**Ni**			0.3817	0.5257
